# Evaluation of the Shaping Ability of Three Thermally Treated Nickel–Titanium Endodontic Instruments on Standardized 3D-printed Dental Replicas Using Cone-Beam Computed Tomography

**DOI:** 10.3390/medicina57090901

**Published:** 2021-08-29

**Authors:** Laura Orel, Oana-Alexandra Velea-Barta, Luminita-Maria Nica, Andreea-Simona Boscornea-Pușcu, Razvan Mihai Horhat, Roxana-Maria Talpos-Niculescu, Cosmin Sinescu, Virgil-Florin Duma, Dan-Dumitru Vulcanescu, Florin Topala, Meda-Lavinia Negrutiu

**Affiliations:** 1 3rd Department, Discipline of Restorative Dentistry and Endodontics, Faculty of Dental Medicine, Victor Babes University of Medicine and Pharmacy, 300041 Timisoara, Romania; orel.laura@umft.ro (L.O.); boscornea-puscu.simona@umft.ro (A.-S.B.-P.); 2 3rd Department, Discipline of Restorative Dentistry and Endodontics, Faculty of Dental Medicine, TADERP Research Center, Victor Babes University of Medicine and Pharmacy, 300041 Timisoara, Romania; horhat.razvan@umft.ro (R.M.H.); roxanaclinci@yahoo.com (R.-M.T.-N.); 3 1st Department, Discipline of Prosthesis Technology and Dental Materials, Faculty of Dental Medicine, Research Center in Dental Medicine Using Conventional and Alternative Technologies, Victor Babes University of Medicine and Pharmacy, 300070 Timisoara, Romania; minosinescu@gmail.com (C.S.); negrutiu.meda@umft.ro (M.-L.N.); 4 3OM Optomechatronics Group, Faculty of Engineering, Aurel Vlaicu University of Arad, 310130 Arad, Romania; duma.virgil@osamember.org; 5 Emergency Hospital for Children Louis Turcanu, 300011 Timisoara, Romania; dannvulcanescu@gmail.com; 6 3rd Department, Discipline of Prosthetic Dentistry, Faculty of Dental Medicine, Victor Babes University of Medicine and Pharmacy, 300041 Timisoara, Romania; ftopala@umft.ro

**Keywords:** 3D-printed dental replicas, endodontic instruments, thermally treated, centring ability, canal transportation, cone-beam computed tomography (CBCT)

## Abstract

*Background and Objectives:* The aim of the present study is to compare the efficacy of three root canal preparation systems in the shaping of 3D-printed root canal replicas of single rooted teeth. *Materials and Methods*: Sixty 3D-printed root canal replicas were produced and divided into three groups, each consisting of twenty samples. Each group was shaped with a different instrument: Reciproc Blue R25/08 (VDW GmbH, Munich, Gemany), WaveOne Gold Primary 25/07 (Dentsply Sirona, Ballaigues, Switzerland), and ProTaper Gold F2 25/08 (Denstply Sirona). To ensure the reproducibility of pre- and post-operative CBCT images of the root canals, the endodontic printed replicas were placed in a mould of silicon impression material. A cone-beam computed tomography (CBCT) software was used to compare pre- and post-instrumentation images collected at three levels of the root canal length: 3, 6, and 9 mm from the apical foramen. The amount of transportation, centring ability, and curvature angle after shaping were evaluated for each system. The results were statistically analysed and compared using one-way analysis of variance (ANOVA). *Results:* Regarding the transportation of the root canal after shaping, significant differences between groups at 3 mm (*p* = 0.010721) and 6 mm (*p* = 0.000046) were recorded in the mesio-distal direction, while in the bucco-lingual significant differences were only observed at 6 mm (*p* = 0.000554). Reciproc Blue removed more dentin from the mesial and buccal wall of the root canal. When evaluating the centring ability of the three systems, significant differences were observed between the groups at the level of 9 mm (*p* = 0.037258) in the mesio-distal direction, and at the level of 6 mm (*p* = 0.038197) in the bucco-lingual direction. Significant differences of the canal curvature angle after shaping were also observed between groups (*p* = 0.000001). Reciproc Blue straightened the curvature the most, while ProTaper Gold the least. *Conclusions:* All systems produced minor root canal transportation. No instrument was able to achieve a perfect centring preparation of the root canal. All systems produced a small degree of root canal straightening.

## 1. Introduction

It is well-known that a straight root canal is an exception rather than a normal situation in human teeth, as most root canals have one or multiple planes curvatures throughout their length [1]. The literature states that root canal curvatures are mostly located in their apical third area [2]. One of the main objectives in shaping the root canal system is to achieve a continuous tapered preparation, while maintaining its original morphology. In narrow and curved root canals, this is still a major challenge in current practice [3].

Additionally, achieving an ideal form after complete root canal shaping is challenging for endodontists and requires a thorough knowledge of the internal anatomy of teeth, of the properties of endodontic instruments, and of their cutting action [4]. The continuous development of nickel–titanium (NiTi) mechanical instruments and the improvements of the alloys’ properties makes current root canal instrumentation easier, faster, and more predictable [5,6,7]. Many shaping NiTi instruments use various structural designs to reduce the operational errors and to achieve predictable root canal preparations. Therefore, analysing the mechanical action of these instruments and of their cutting efficiency is important in order to develop and simplify the endodontic instrumentation step [8]. 

There is no specific procedure for an effective comparison of root canal shaping in a reproducible manner, even though there are numerous studies detailing various in vitro methods for evaluating the efficacy of instruments [9]. Although some researchers have recommended the use of extracted teeth for this purpose, for a more reproducible clinical situation, involving dentine properties, others concluded that the benefits of a high standardization using simulated artificial root canals is more precise, and outweighs their disadvantages [9].

Using the 3D-printing process, standardized endodontic tooth replicas with a certain shape and size of the simulated root canals can be obtained, taking into consideration that most of the foramen diameters vary between 0.20 and 0.29 mm [10]. Unlike transparent resin blocks which can be evaluated only by using photographs taken before and after the procedure, endodontic replicas can be printed from radiopaque materials. This allows the use of more modern and precise imagistic investigation tools, such as cone-beam computed tomography (CBCT) [9].

CBCT is a modern and non-invasive method of diagnosis, with low radiation dose, which allows for the evaluation of detailed images using different settings. It is used to compare the anatomical structures of the root canal system before and after bio-mechanical shaping, allowing the detection of deviations and transport, centring ability, and degree of curvature before and after the preparation [11]. CBCT images are highly accurate compared to traditional techniques, have excellent reproducibility, and can provide several images of a single canal at different levels and in different planes [12].

Incorporating CBCT in endodontic clinical practice as an additional resource for the diagnosis, treatment planning, and for follow-ups has significantly contributed to establishing more efficient working protocols [13]. CBCT images manage to identify both anatomic and pathologic alterations of the dental structures, and they have been shown to reduce the incidence of false-negative results [14]. Furthermore, CBCT is still the most reliable method used to assess root canal anatomy and morphology before and after treatment, especially regarding apical transportation and centring abilities of the shaping systems [15].

This is the reason the aim of the present study is to assess the efficiency of endodontic shaping techniques on standardized dental replicas, using two systems with reciprocating motion: Reciproc Blue (RB) (VDW GmbH, Munich, Germany) and WaveOne Gold (WOG) (Dentsply Sirona, Ballaigues, Switzerland), and one using continuous rotation motion, ProTaper Gold (PTG) (Dentsply Sirona), through pre- and post-operative CBCT imagistic techniques. The tested null hypothesis was that all the three studied NiTi systems have similar shaping characteristics and centring abilities in the root canal preparation.

## 2. Materials and Methods

### 2.1. Design and Printing of the Dental Replicas 

A standardized design of a single rooted dental replica with an ideal simulated root canal was created using a special 3D-printing programme, Autodesk Fusion 360 (San Rafael, CA, USA). All the standardized simulated root canals had an 18 mm working length, an apical diameter of 0.20 mm, and a 2% taper along their length, with a moderate curvature located in the apical third and an angle of 18 to 20°, as most natural teeth are curved in the apical third, and a curvature angle between 5 and 20° is considered moderate [2]. 

The dental replicas were printed with a Prusa SL1 3D printer (Josef Prusa, Prusa Research, Prague, Czech Republic), in 25 µm thick layers using stereo-lithography (SLA), from Esun Standard White resin (Esun, Shenzen, China).

**Figure 1:**
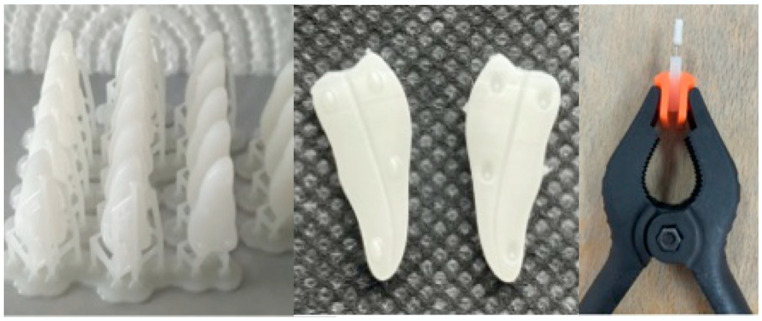
Dental replicas after the printing process. The two halves of the printed dental replica with the simulated curved root canal. Sticking of the two halves of the dental replica.

As the objective was to obtain replicas with narrow and curved canals, several attempts were made to print them. Unfortunately, after the first final polymerization of the resin, the simulated root canals were partially obstructed and not visible in the final 3 mm of the apical part. Therefore, a dental replica made from two separate pieces which were later interlocked was developed (Figure 1).

The sticking of the two halves was achieved by maintaining a 0.15 mm K-file inside the simulated root canal. After positioning, the two pieces were tightly held together with a clamp to make sure that they were interlocked, and after the resins’ curing, the K-file was removed for the endodontic instrumentation procedure (Figure 1).

Sixty printed dental replicas divided into three groups, each with twenty replicas (*n* = 20) were included in this in vitro study.

### 2.2. Preoperative CBCT Scanning

The CBCT analysis of the replicas was performed using the SOREDEX CRANEX^®^ 3D device (SOREDEX, PaloDEx Group Oy, Tuusula, Finland), an imagistic system that offers high quality images, with panoramic, cephalometric, and endodontic programs. The 3D images were automatically engineered in DICOM format, the device being provided with a specific software for each specific situation [16]. To be able to standardize the CBCT measurements, a conformer made of Optosil Comfort Putty impression material (Kulzer GmbH, Hanau, Germany) was made. The conformer was in the shape of a mandible observed from a frontal norm. The dental replicas were placed in the same position before and after instrumentation, with the root canal foramen oriented towards the distal side, therefore the external part of the canals’ curvature corresponded to the mesial side of the replica.

**Figure 2:**
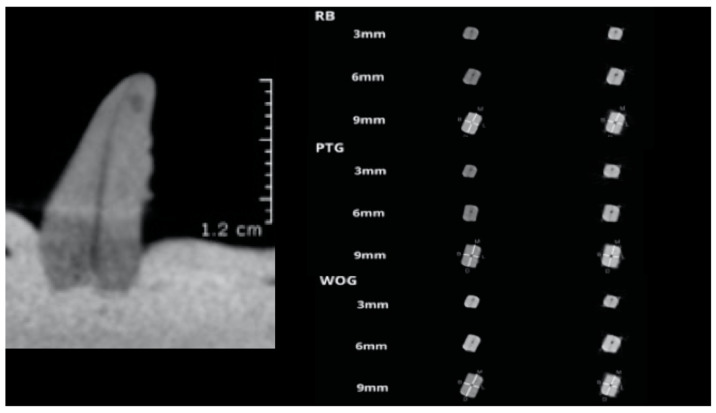
CBCT image of a printed dental replica with a simulated curved root canal, and RB (Reciproc Blue) PTG (ProTaper Gold) and WOG (WaveOne Gold) pre- and post-instrumentation aspects at different levels.

The teeth were mounted in an upside-down position, with the coronal part embedded in the impression material, while their roots were out of it. This position was preferred to ensure a clear radiological image of the root canal, without the impression material influencing the quality of the CBCT image (Figure 2). 

### 2.3. Shaping of the Dental Replicas 

The shaping of the samples was completed by a single operator. As the canals of the dental replicas had an apical diameter larger than 0.15 mm, no hand negotiation and glyde path management was necessary. 

Shaping was achieved with thermally treated NiTi endodontic instruments with the same tip diameter 0.25 mm, and with similar taper. The first group was shaped with the ProTaper Gold system (Dentsply Maillefer, Ballaigues, Switzerland) in the sequence S1-S2-F1-F2 as recommended by the producers, using an endodontic motor X-Smart Plus (Dentsply Sirona) in continuous rotation at 300 rpm and corresponding torque settings, until the F2 25/08 instrument reached the working length. Group II was shaped with Reciproc Blue (VDW GmbH, Munich, Germany) instrument RB25 with a 0.25 mm tip diameter and an 8% taper on the first 3 mm in reciprocating motion using the same motor. Group III underwent shaping with the WaveOne Gold system (Dentsply Maillefer, Ballaigues, Switzerland), instrument WaveOne Gold Primary, with a tip diameter of 0.25 mm and a 7% taper. During shaping, copious irrigation with alcohol was used after each instruments’ passage into the root canal and recapitulation with a hand K-file 0.10 mm after each file was performed, to avoid canal blockage. 

### 2.4. Postoperative CBCT Scanning 

Postoperative scanning underwent the same protocol as the preoperative scanning. The mesio-distal and bucco-lingual canal deviation, the centring ratio (at 3 mm from the apical foramen, at 6 mm, and at 9 mm), and the curvatures’ angle were analysed (Figure 2). The CBCT images were evaluated to assess the centring ability of the instruments and the root canal transportation, using the methods described by Gambill et al. [17].

For **the root canal transportation**, the following equations were used:

(M1 − M2) − (D1 − D2) (for the mesio-distal direction) (1)

(B1 − B2) − (L1 − L2) (for the bucco-lingual direction)(2)

where M1 is the smallest distance from the mesial root surface to the root canal contour before preparation; D1 is the smallest distance from the distal root surface to the root canal contour before preparation; M2 is the smallest distance from the mesial root surface to the periphery of the canal after instrumentation; D2 is the smallest distance from the distal root surface to the periphery of the canal after instrumentation; B1 is the smallest distance from the buccal root surface to the canal contour before instrumentation; B2 is the smallest distance from the buccal root surface to the periphery of the canal after instrumentation; L1 is the smallest distance from the lingual root surface to the canal contour before preparation; and L2 is the smallest distance from the lingual root surface to the periphery of the canal after preparation.

According to these formulas, a difference equal to zero means no root canal transportation, whereas positive values show mesial or buccal transportation, and negative values show distal or lingual transportation.

**The centring ability** of each instrument at each analysed level was determined by using the following ratio:

(M1 − M2)/(D1 − D2)(3)

(B1 − B2)/(L1 − L2)(4)

The fraction with the smaller value was selected for the statistical analysis. The mean centring ratio reflects the capacity of the instruments to be kept centred in the canal. According to these formulas, a value equal to 1 represents a complete centring, whereas other values show changes in the canal pathway [18].

The angle of curvature of the root canals was measured before and after instrumentation according to the Schneider’s method [19]. The difference of the initial/final degrees was recorded and then compared.

On the CBCT exam, the root canal curvature was measured using the OnDemand 3D software (OP 3D™ Pro, KaVo Dental GmbH, Biberach, Germany) following the method previously described. Thus, two straight lines were used: the first starts from the canal orifice level, and follows the direction of the root canal up to the point where it starts to deviate; the second starts from the apical foramen, and is connected to the same deviation point. The angle formed at the intersection of the two lines was measured and referred as angle of the curvature [2,19,20].

## 3. Results

### Statistical Analysis

The statistical analysis of the data was performed using Microsoft Excel and SPSS 22.0 (SPSS Inc., Chicago, IL, USA). Each set of measurements was analysed using the Kolmogorov–Smirnov test. Statistical significance level was set at *p* < 0.05. Descriptive statistics was provided for all data sets. For comparison between data sets, the ANOVA test was performed.

### 3.1. Canal Transportation

#### 3.1.1. Mesio-Distal Transportation

**Figure 3:**
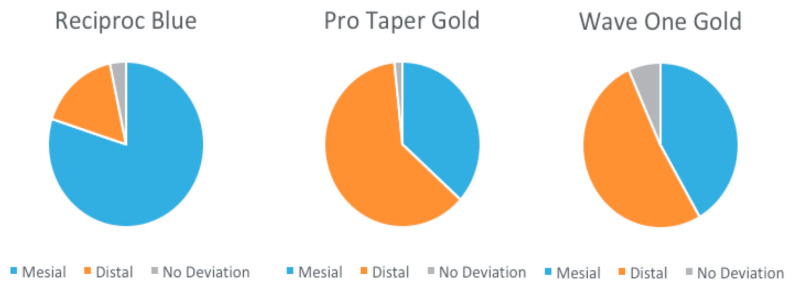
Direction of the mesio-distal transportation for Reciproc Blue, ProTaper Gold, and WaveOne Gold.

All the studied instruments had a positive median, indicating that they all induced mesial transportation. A descriptive analysis regarding the mesio-distal transportation is presented in Table 1. Statistically significant differences between were found between the groups at levels of 3 mm (*p* = 0.010721) and of 6 mm (*p* = 0.000046). At 3 mm, RB removed more resin compared to the other systems, with a mean of 0.0935 mm, while the mean was only 0.0520 mm for WOG and 0.0410 mm for PTG. At 6 mm, the highest mean value was recorded for PTG, i.e., 0.1585 mm, while the lowest was recorded for RB, with a mean of 0.0525 mm. At the level of 9 mm, no statistically significant differences could be observed between the different instruments, as *p* = 0.057590 > 0.05. The direction of mesio-distal transportation for each study group is represented in Figure 3.

#### 3.1.2. Bucco-Lingual Transportation

**Figure 4:**
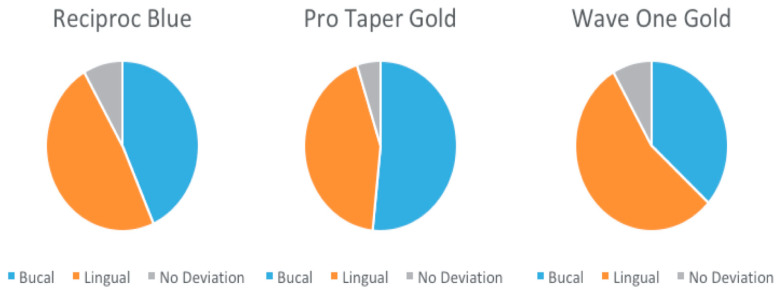
Direction of bucco-lingual transportation for Reciproc Blue, ProTaper Gold, and WaveOne Gold.

The median values of all the three systems included in the present study were positive, indicating that transportation occurred in a buccal direction, according to the present analysis (Table 1). Significant differences between the groups at the level of 6 mm (*p* = 0.000554) were recorded, with Reciproc Blue registering the highest mean value, equal to 0.0845 mm. No statistically significant differences could be observed at the level of 3 mm, as *p* = 0.225694 > 0.05, nor at the level of 9 mm, as *p* = 0.132043 > 0.05 between the three systems. The direction of bucco-lingual transportation for each group is represented in Figure 4.

### 3.2. Centring Ability

There was no system in the present study with a median value equal to unit, indicating that no perfect centring ability was achieved in the mesio-distal direction for the tested instruments (Table 1). There were clear differences between the groups only at the level of 9 mm (*p* = 0.037258), where WOG registered the lowest mean values, showing the lowest centring ability at this level, followed by PTG and RB. No statistically significant differences could be observed at the level of 3 mm, as *p* = 0.916488 > 0.05, nor at the level of 6 mm, as *p* = 0.084446 > 0.05. Results of the ANOVA test are presented in Table 2.

The centring ability in the bucco-lingual direction revealed that none of the systems under consideration had a median value equal to the unit, indicating that no system had a perfect centring ability.

Significant differences between the groups were recorded at the level of 6 mm (*p* = 0.038197), where RB registered the lowest values in comparison with WOG and PTG, meaning that RB had the lowest centring capacity. No statistically significant differences could be observed at the level of 3 mm, as *p* = 0.502930 > 0.05, nor at the level of 9 mm, as *p* = 0.442457 > 0.05. All systems recorded similar means of 0.7550–0.7920 mm at the level of 3 mm, and 0.7842–0.8538 mm at the level of 9 mm. Results of the ANOVA test are presented in Table 2.

### 3.3. Canal Curvature

**Figure 5:**
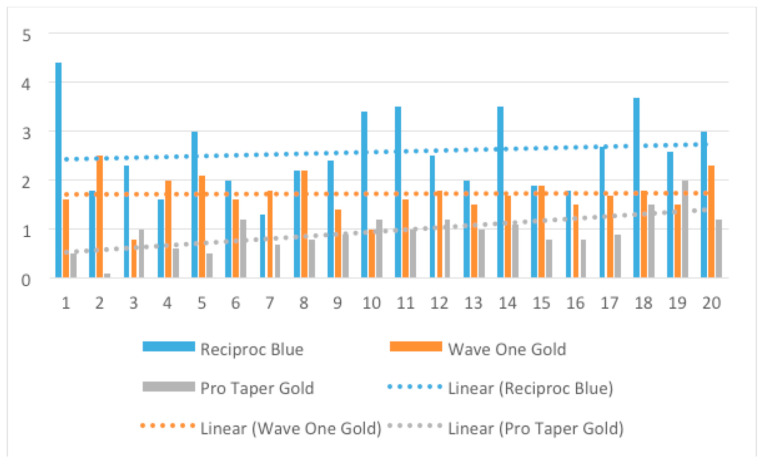
Values and trend line of canal curvature angle after shaping with WaveOne Gold, Reciproc Blue, and ProTaper Gold.

A descriptive analysis regarding the canal curvature is presented in Figure 5. A minor decrease in the curvature angle was registered for all instruments. The mean difference between the initial and the final angle was 0.95° for PTG, 1.72° for WOG, and 2.58° for RB. As it can be observed, RB induced the highest modification of curvature angle in comparison with WOG and PTG. This means that RB straightened more the root canal path than WOG and PTG. 

## 4. Discussion

The aim of the present study was to compare the shaping ability of three newly introduced NiTi endodontic instruments. They are frequently used in daily clinical practice for an accurate shaping of curved root canals. Two of them use reciprocation motion (Reciproc Blue and WaveOne Gold), while the other uses a continuous rotation (ProTaper Gold). Canal transportation, centring ability at 3 mm, 6 mm, and 9 mm distance from the apex, as well as modifications of the angle of root canals curvatures on endodontic printed replicas were analysed for each system, both in the mesio-distal and in the bucco-lingual direction.

The aim of root canal shaping is to clean and shape the endodontic system while maintaining its original anatomy, allowing in the end for a three-dimensional obturation [3]. Curved root canals are still challenging for practitioners, as all instruments and shaping techniques have been shown to affect the shape of the root canal one way or another [5,6,21].

Apical transportation and centring ability of the endodontic instruments have been evaluated using a variety of methods [22], but CBCT is one of the most often used as it is repeatable and enables for specimen protection [23]. This radiologic investigation allows the acquisition of several images of the root canal before and after instrumentation. This offers accurate information about the root canal anatomy and about its modification during endodontic procedures [24,25].

In in vitro studies, the use of resin blocks and extracted natural teeth is more common among the options available for evaluating the root canal shaping [26]. Standardization of the root canal diameter, length, and curvature in terms of angle and radius, standardization of the research method, and exclusion of parameters that could influence the preparation outcome, as well as high credibility as an ideal experimental model for the analysis of endodontic shaping technique, are advantages of simulated root canals in resin blocks [27]. However, one significant issue with resin blocks is that they have different mechanical properties than natural dentin. The micro-hardness of the dentin around the pulp, for example, is twice that of resin blocks. This indicates that, in natural teeth, higher stress values are given to instruments during root canal instrumentation than in resin blocks [21]. Additionally, the heat generated by the mechanical instrumentation can soften the resin to the point where it clutches the cutting blades of the instruments. Caution should be exercised when extrapolating these findings to clinical cases [28].

Furthermore, the resin blocks have particles of larger size than dentin. As a result, they may obstruct the insertion and operation of the endodontic instruments by blocking the root canals [21]. Additionally, resin blocks can only be evaluated through CBCT imagistic methods by inserting some contrast agents such as calcium hydroxide or barium sulphate in the simulated root canals, method used by Mathew et al. in a similar in vitro study [27].

On the other hand, while extracted natural teeth almost perfectly mimic the micro-environment of root canal preparation in clinical setting, the lack of standardization of teeth regarding the apical patency, diameter of the foramen, and the angle of curvature are significant disadvantages for their use in research studies. CBCT imaging technique may provide reliable results regarding root canal shaping evaluation, to extrapolate the obtained results to current clinical practice [21].

Regarding root canal transportation, two methods are available to perform measurements on CBCT scans. While some research protocols found in the literature used superimposed pre- and post-operative images to evaluate the changes in the root canal path due to preparation [29], others used pre- and post-operative cross-sectional pictures at three different levels to assess the distance between the external root surface and internal canal wall from mesial and distal aspects. Changes in the root canal path are then calculated using applicable formulas. This method was also used in the present study to detect changes in the apical, middle, and coronal thirds of the root canals by measuring the distance between the external root surface and the internal root canal wall at three levels (3, 6, and 9 mm from the apex) before and after the instrumentation [29].

The present study combined the advantages of the standardized method offered by using endodontic resin blocks [30] with the advantages of the CBCT imagistic investigation by evaluating standardized printed dental replicas made of a radio-opaque resin [31]. This way, on the CBCT images of the 3D-printed specimens, a clear difference could be observed between the canal contour and the outer root walls, so instrumentation measurements before and after could be taken precisely.

Based on the obtained results, the null hypothesis was rejected. Differences were recorded in shaping between the three tested systems both in mesio-distal and bucco-lingual directions, with those in mesial direction being the most significant, especially for Reciproc Blue. These results are in accordance with a previous study developed by Mamede-Neto et al., which concluded that all investigated systems produced mesial and buccal transportation, with significant differences only for Reciproc in the mesio-distal direction, and for Reciproc and ProTaper Gold in the bucco-lingual one [32]. In comparison with [32], the present study revealed statistically significant differences between all three investigated systems at 3 and 6 mm in the mesial direction and at 6 mm in the buccal direction. 

Although canal transportation was induced during the cutting action for the enlargement of the root canal, the shape of the simulated root canals was not significantly modified from the original trajectory. These results are similar with other studies on human teeth regarding canal transportation and centring ability of NiTi instruments, which concluded that they tend to preserve the natural shape of the root canals [33,34].

In another study of Keskin et al., at the halfway point between the canal orifice and the beginning of the curve, the apex of the curve, and the terminus of the canals, the canal transportation values for WaveOne Gold were much lower than those for Reciproc blue. At the canal orifice and at the beginning of the curve, on the other hand, significant variations between instruments were noticed [35].

The present study revealed statistically significant differences in the mesio-distal direction at 3 mm, where Reciproc Blue removed more resin from the mesial side of the replica, therefore from the external part of the curvature of the root canals, in comparison to WaveOne Gold and ProTaper Gold. At the level of 6 mm, the middle third of the root canal, the results confirmed that ProTaper Gold removed more resin from the mesial side than Reciproc Blue and WaveOne Gold. 

Regarding the bucco-lingual canal transportation, the present study revealed significant differences between the tested instruments only at 6mm from the apex, where Reciproc Blue had a greater degree of transportation than both WaveOne Gold and ProTaper Gold. Additionally, WaveOne Gold was the system that had the least centred preparation at 9mm in mesio-distal direction, whereas in bucco-lingual direction, at 6 mm from the apex, Reciproc Blue 25 had the least centred preparation compared to WaveOne Gold Primary and ProTaper Gold F2. 

As clinical impact of canal transportation, Wu et al. reported that apical transport can affect root canal cleanliness, disinfection, and sealability [36]. However, Peters (2004) showed that only transportation of more than 0.3 mm has an important impact on the long-term prognosis of endodontic treatment, while values of up to 0.15 mm are considered acceptable [37]. As the highest mean values recorded in the present study for the canal transportation were of 0.2555 mm in mesio-distal direction, and of 0.0845 mm in the bucco-lingual, therefore in acceptable limits, they can be considered with no major impact on the predictability of the performed treatment, according to the mentioned study.

Regarding the degree of canal straightening following the shaping of the simulated root canals, Reciproc Blue recorded the highest differences between the initial and final curvature angle, while ProTaper Gold recorded the lowest such differences. Thus, the latter system modified the least the angle of curvature. Data obtained in the present study, after evaluating the three endodontic shaping systems included in this research, revealed minor mean values of the curvature angle variation ranging from 0.95° to 2.58°. These results are comparable to those of other investigations in which the experimental studies were conducted under similar settings as in the present one [34]. However, no comparison was found in the literature between the three shaping systems included in the present study. 

According to the findings of this work, all three endodontic shaping systems caused acceptable canal transportation in the apical, mesial, and coronal third of the root canal, as well as a minor straightening of the curvature and a small deviation in terms of assessing the centring ability of each instrument. These findings are in accordance with data found in the literature that demonstrate thermally treated endodontic rotary or reciprocating NiTi instruments induce no considerable shaping mishaps and errors, possess better centring ability, and produce less transport in comparison to conventional or even M-wire NiTi instruments [38,39,40]. 

### Limitations

The current study used a rather small number of printed teeth in each group (20 teeth/group), but it was statistically significant to examine and compare the results. Additionally, the use of the micro-CT method of investigation might provide more accurate results.

## 5. Conclusions

Root canal dental replicas made using stereo-lithographic 3D-printing allow researchers to create different root canal configurations for methodical studies of the advantages and limitations of root canal instruments. Within the limitations of this study, all systems produced root canal transportation. No file system achieved perfect centring ability of root preparation. All the three tested systems produced minor straightening of the root canal.

1. Reciproc Blue induced more significant mesial and buccal root canal transportation than WaveOne Gold and ProTaper Gold;
2. The centring ability was lower for WaveOne Gold in the mesio-distal direction, and for Reciproc Blue in the bucco-lingual direction;
3. Reciproc Blue recorded the highest value for straightening the angle of curvature following the shaping of the simulated root canals, while ProTaper Gold was the system that changed the angle of curvature the least;
4. ProTaper Gold shaping system showed a more anatomic pattern of preparation with less root canal transportation, a better centring ability, and a lower tendency to straighten the curvature of the simulated root canal.

## Figures and Tables

**Table 1:** The mean values (mm) and the standard deviation (SD) for the mesio-distal and bucco-lingual transportation and centring ability at the three considered levels, and the variation of the canal curvature angle (°).

Level of Investigation	System (*n* = 20/Group)	Mesio-Distal Transportation	Bucco-Lingual Transportation	Mesio-Distal Centring	Bucco-Lingual Centring
		Mean ± SD			
3 mm	Reciproc Blue (RB)	0.0935 ± 0.0873 *	0.0525 ± 0.0363	0.6901 ± 0.1741	0.7550 ± 0.1447
	Wave One Gold (WOG)	0.0520 ± 0.0329	0.0395 ± 0.0157	0.6886 ± 0.1587	0.7920 ± 0.0912
	ProTaper Gold (PTG)	0.0410 ± 0.0255	0.0495 ± 0.0161	0.7072 ± 0.1344	0.7601 ± 0.0674
6 mm	RB	0.0525 ± 0.0414	0.0845 ± 0.0465 *	0.7624 ± 0.1802	0.6124 ± 0.1392 *
	WOG	0.0950 ± 0.0960 *	0.0390 ± 0.0460	0.7029 ± 0.2745	0.7535 ± 0.1939
	PTG	0.1585 ± 0.0579 *	0.0380 ± 0.0257	0.6198 ± 0.1063	0.7025 ± 0.1779
9 mm	RB	0.1770 ± 0.0628	0.0295 ± 0.0302	0.5547 ± 0.1137	0.8378 ± 0.1515
	WOG	0.2555 ± 0.1518	0.0310 ± 0.0524	0.4185 ± 0.2226 *	0.8538 ± 0,2242
	PTG	0.2405 ± 0.0877	0.0570 ± 0.0564	0.5113 ± 0.1441	0.7842 ± 0.1522
		Mean ± SD			
Canal	RB	2.58 ± 0.8115 *			
curvature	WOG	1.72 ± 0.4043			
angle	PTG	0.95 ± 0.4020			

* Statistically significant differences between groups.

**Table 2:** The *p*-values of the ANOVA statistical analysis between the considered groups regarding the centring ability at the three investigated levels.

Levels of Investigation	Mesio-Distal Centring Ability *p*-Value	Bucco-Lingual Centring Ability *p*-Value
3 mm	0.916488	0.502930
6 mm	0.084446	* 0.038197
9 mm	* 0.037258	0.442457

* Statistically significant differences between groups.

## Data Availability

Data is available upon request from the corresponding author.
